# A physics-driven workflow for gas-sand identification in Pliocene turbidites using pre-stack inversion and seismic attributes, offshore Egypt

**DOI:** 10.1038/s41598-025-31461-9

**Published:** 2026-01-11

**Authors:** Ali Mahdy, Ahmed Helmi, Ahmad Sobhy Helaly, Abdullah M. E. Mahmoud

**Affiliations:** 1 Department of Geophysics, Faculty of Science, Ain Shams University, Cairo, Egypt; 2 Shell Egypt, Fifth Settlement, Cairo, Egypt

**Keywords:** Rock-physics, Simultaneous inversion, Geo-body extraction, Energy science and technology, Solid Earth sciences

## Abstract

This study presents a physics-driven workflow that integrates pre-stack simultaneous inversion of P-impedance, S-impedance, and density with multi-attribute analysis and geo-body extraction to resolve thin, isolated gas-sand channels in the compartmentalized Pliocene turbidite system of the Sapphire Field, offshore Nile Delta, Egypt. Unlike conventional post-stack inversion or AI-based bright-spot detection, our approach leverages rock-physics-guided cross-plotting (V_p_/V_s_ vs. P-impedance), validated by blind-well testing, to achieve robust lithology–fluid discrimination under sparse well control. Gas-sand facies are reliably identified by low P-impedance (< 18 (m/s)·(g/cm^3^)) and Vp/Vs ratios (< 1.65), while gradient magnitude and variance attributes delineate channel edges and fault-related compartmentalization with high fidelity. Critically, the workflow overcomes thin-bed resolution limitations through elastic trend analysis rather than absolute layer thickness, offering a transferable methodology for similar clastic deepwater plays worldwide. However, uncertainties persist in ultra-thin beds (< 9 m) due to seismic bandwidth constraints (~ 10–60 Hz), and inversion reliability depends on accurate low-frequency modeling and angle-stack quality. By bridging first-principles rock physics with high-resolution seismic attributes, this study advances quantitative interpretation and delivers actionable insights for exploration risk reduction and optimal well placement

## Introduction

Effective subsurface characterization is critical for hydrocarbon exploration in complex, compartmentalized depositional systems like the Pliocene turbidites of the Nile Delta. Conventional seismic interpretation—reliant on qualitative amplitude analysis—often fails to resolve thin gas-sand channels or distinguish lithology from fluid effects. To overcome these limitations, we integrate pre-stack seismic inversion with rock-physics modeling and multi-attribute analysis, enabling quantitative prediction of reservoir properties directly from seismic data^1^.

This study proposes an innovative methodology that transcends these limitations by integrating established seismic data interpretation and inversion techniques. This integrated approach fosters a more comprehensive understanding of the subsurface, enabling a more robust assessment of potential hydrocarbon reservoirs in the area. The proposed workflow not only enhances the accuracy of reservoir characterization but also reduces exploration uncertainties, ultimately leading to informed decision-making in hydrocarbon exploration and development.

Cross-plotting has become a fundamental technique in rock physics analysis because of its ability to rapidly and visually depict relationships among different attributes^2^. In cross-plot spaces, distinct clusters often form for various lithologies and fluid types, facilitating a straightforward interpretation^3^.

Rock-physics cross-plotting—pioneered by Pickett^4^ and Goodway et al.^5^—enables visual discrimination of lithology and fluid content through key elastic parameters: P-impedance, Vp/Vs ratio, Lambda-rho (λρ), and Mu-rho (µρ). P-impedance integrates density and P-wave velocity to reflect both matrix and fluid properties,Vp/Vs is highly sensitive to gas saturation; λρ serves as a robust fluid indicator; and µρ primarily responds to lithological variations^3,6^. Seismic attributes, quantitative measurements derived from seismic data, provide valuable insights into the subsurface beyond the traditional seismic reflection amplitude^7^.

To enhance the characterization of the highly compartmentalized Pliocene turbidite reservoir in the Sapphire Field (offshore Mediterranean, Egypt), we integrate simultaneous pre-stack inversion results with a suite of seismic attributes—quantitative measures derived from 3D seismic data that highlight geological features beyond raw amplitude. Variance is used to map fault zones and channel margins by detecting trace-to-trace discontinuities; gradient magnitude accentuates sharp changes in seismic waveform associated with structural and stratigraphic boundaries; instantaneous phase reveals depositional continuity independent of amplitude variations; and RMS amplitude provides a measure of relative seismic energy, which—when calibrated to well data—helps identify reservoir-prone intervals^8^. All attributes are interpreted within a rock-physics framework and validated against blind wells to ensure geologically consistent and robust reservoir delineation, particularly in areas with sparse well control^9^.

By integrating and interpreting a suite of seismic attributes, geoscientists can gain a more comprehensive understanding of subsurface geology, leading to improved exploration and development strategies.

In recent years, there has been a growing emphasis on integrating advanced seismic inversion techniques with seismic attributes for reservoir characterization. Simultaneous inversion leverages multi-angle/offset data, providing high vertical and lateral resolution necessary for resolving thin beds and complex impedance contrasts in features like top-channel sands. Simultaneous inversion consistently outperforms post-stack and partial-stack inversion methods in improving resolution, integrating seismic datasets, and characterizing complex geological features like top-channel sands and base-gas reflectors. Key studies have demonstrated these benefits in clastic reservoirs^10,11^. For example, post-stack inversion struggled to resolve sand-shale differentiation, but simultaneous inversion successfully distinguished lithologies using P-impedance and V_p_/V_s_ properties. Post-stack methods lack sensitivity to elastic variations and fail to resolve thin-bedded sands or distinguish lithology-fluid differences^12,13^.

Pre-stack simultaneous inversion, which inverts multiple seismic stacks simultaneously, offers a significant advantage over traditional post-stack or pre-stack inversions by providing a more comprehensive picture of the subsurface elastic properties^14^**.** This comprehensive picture, when combined with a suite of seismic attributes sensitive to lithology and pore fluid content, allows for a more robust approach to reservoir facies identification^15^. By leveraging the strengths of both pre-stack simultaneous inversion and seismic attributes, this study aims to delineate and characterize reservoir facies, ultimately leading to improved exploration and development strategies.

## Geological setting

**Fig. 1:**
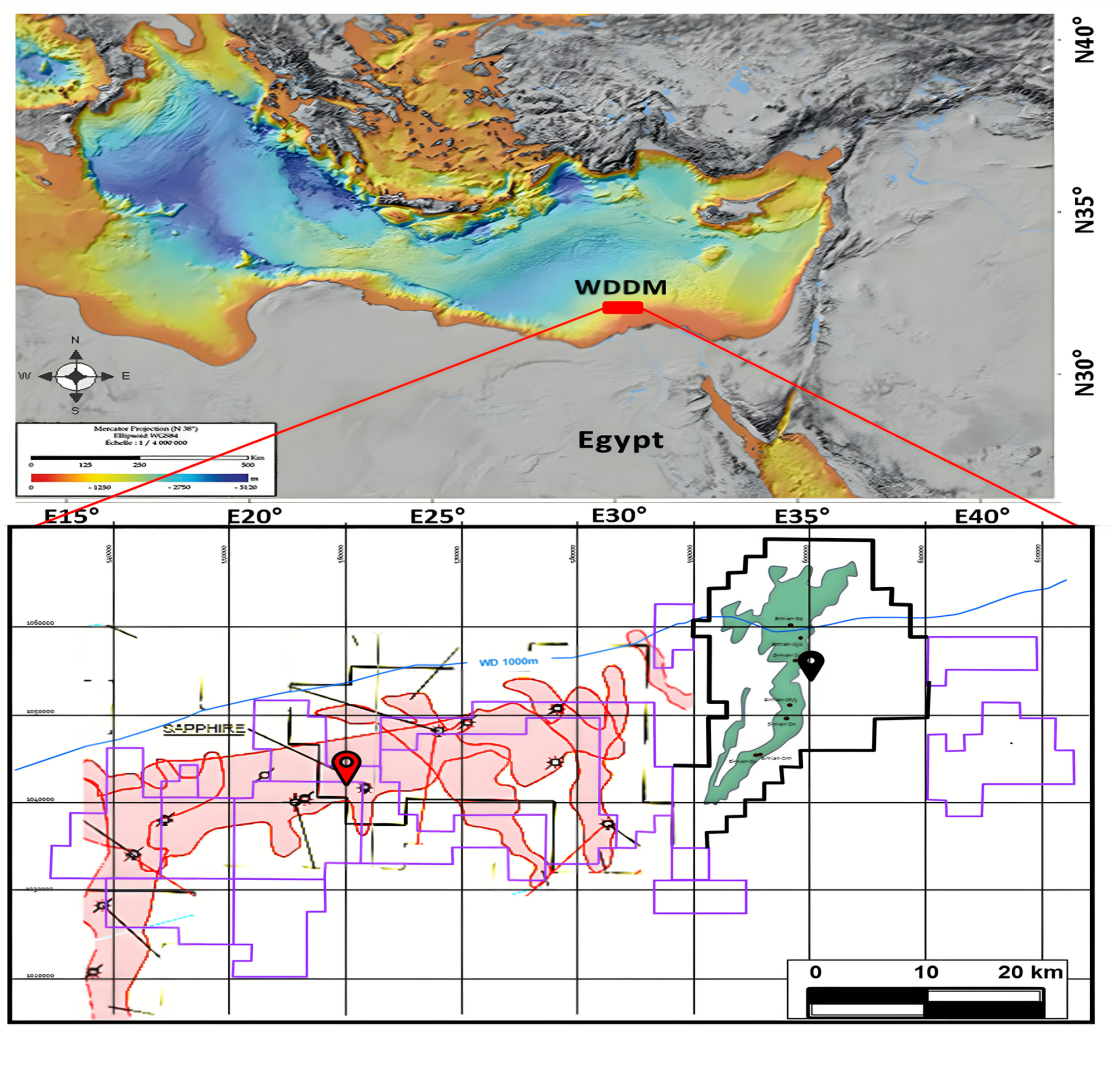
Illustrates the location map of the West Delta Deep Marine concession, where the red location label indicates the Sapphire Field, while the black label indicates the Simian Field.

Sapphire Field, a gas-bearing Pliocene reservoir, is nestled within the West Delta Deep Marine (WDDM) concession, roughly 120 km northeast of Alexandria, Egypt (Fig. 1). Extending approximately 35 km in the ENE-WSW direction, the field sits at water depths between 250 and 700 m (EGPC, 2023). Sapphire sands, constituting part of the Lower Pliocene Kafr-El-Sheikh Formation, comprise a layered sequence of gas-bearing sandstones and claystones ^16,17^.

**Fig. 2:**
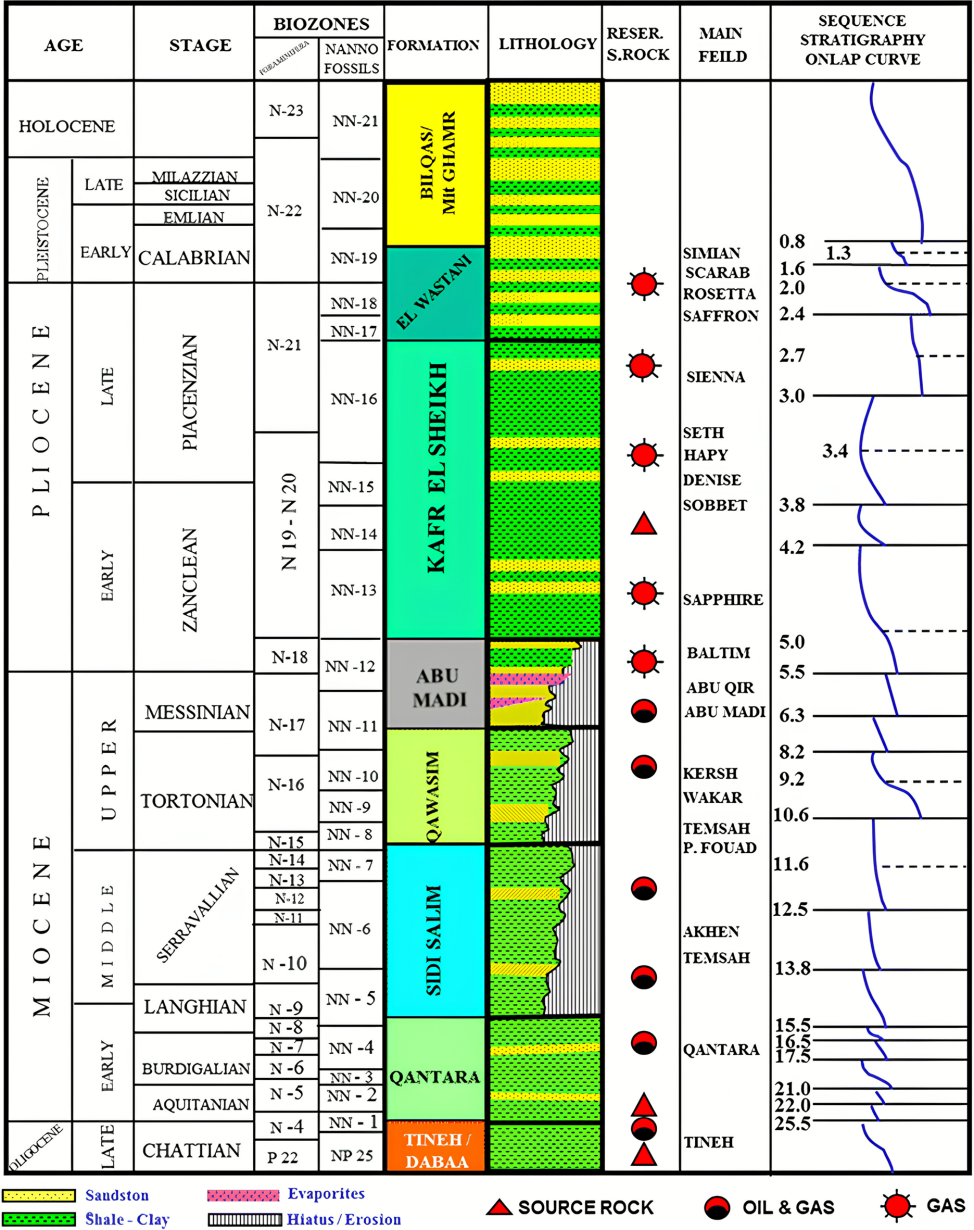
Presents the Nile Delta stratigraphic column and hydrocarbon system, showing the location of the Sapphire Field at the base of the Kafr El-Sheikh Formation ^20^.

The Sapphire Field (Fig. 1) is classified as a subsurface delta slope channels system, featuring intricate turbiditic channel and lobe reservoirs at the base of the Lower Pliocene Kafr El Sheikh Formation (Fig. 2)^18^. One of the primary challenges in field development involves comprehending the distribution of thin sandstone beds and heterolithic facies, which are believed to play a crucial role in facilitating the connectivity between the main sandstone bodies^16,17^.

### Location of study area

The West Delta Deep Marine (WDDM) concession, situated approximately 90 km off the Egyptian coast in the eastern Mediterranean, lies on the northwestern boundary of the Nile Delta (Fig. 1). Its geographic coordinates are between 32°02′52″N and 32°06′15″N in latitude and 30°17′12″E to 30°43′55″E in longitude, encompassing ocean depths ranging from 200 to 1200 m (EGPC, 2023).

## Methodology

**Fig. 3:**
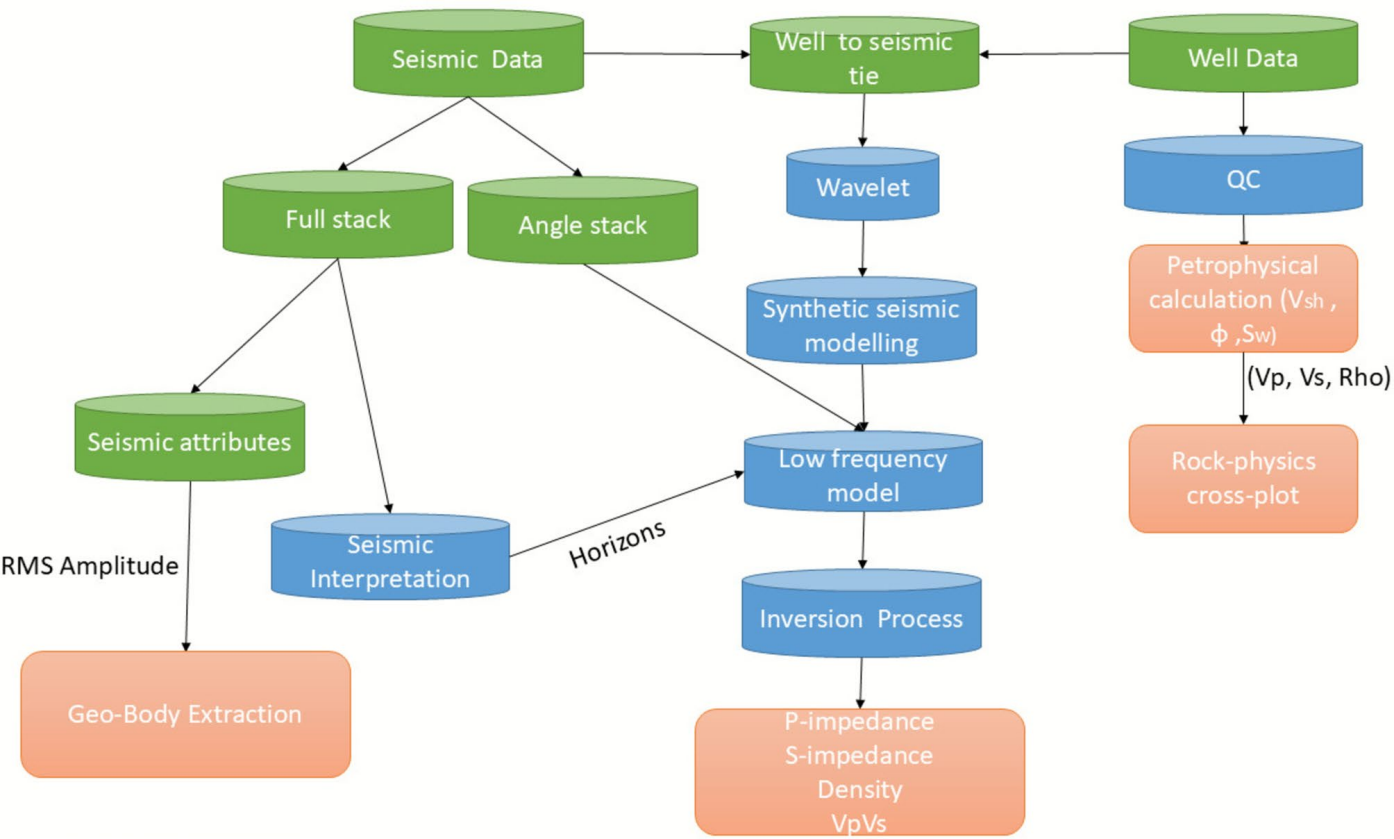
Illustrates the workflow applied for the area of study, encompassing the key stages of data type, data preparation, seismic interpretation, and inversion and reservoir characterization.

The proposed workflow (Fig. 3) integrates four sequential stages: data acquisition and type selection, data preparation, seismic interpretation, and inversion-based reservoir characterization—designed specifically for thin-bedded, compartmentalized turbidite systems with limited well control.

### Data type

*Seismic data*: The specific acquisition method for offshore and the processing configuration are determined based on the exploration objectives and subsurface conditions.

*Well data*: Well data, including well logs (sonic and density) and well markers (tops and horizons), were incorporated into the workflow. Well logs provide crucial information about subsurface lithology, porosity, and fluid properties, which serve as ground truth for seismic data calibration and interpretation.

### Data preparation

The 3D seismic survey over the Sapphire Field (offshore Mediterranean, Egypt) was reprocessed in 2014 using pre-stack depth migration and converted to time for interpretation. To enable pre-stack simultaneous inversion, angle stacks were generated in four incident-angle ranges—near (0°–20°), mid (20°–30°), far (30°–40°), and ultra-far (40°–50°)—selected to optimize sensitivity to P-impedance, S-impedance, and density, respectively^19^. A deterministic wavelet was extracted by deconvolving the seismic trace at well locations using the reflectivity series derived from sonic and density well logs. The wavelet was assigned a constant phase rotation of –79°, determined through iterative phase testing to maximize alignment between synthetic and real seismic traces, achieving a correlation coefficient of 0.913. Wavelet stability across the survey was verified using RMS amplitude maps and spectral quality control (Fig. 2, confirming consistent bandwidth (10–60 Hz. A low-frequency model (< 10 Hz was constructed by kriging well-derived impedance logs, constrained by regional velocity trends from check-shot surveys, to anchor absolute impedance estimates below the seismic bandwidth. Quality control revealed elevated noise in ultra-far angles (> 40° due to attenuation and multiples; these stacks were tapered and smoothed prior to inversion to preserve signal fidelity. This integrated data preparation ensures robust, well-calibrated inputs for elastic property inversion.

Integrating well log data with seismic data is essential for calibrating the inversion. Well logs provide ground truth for elastic properties, allowing for the tuning of the inversion parameters and improving the accuracy of the results. The observed stronger peak amplitudes at the base of the reservoir could indeed be influenced by factors such as higher porosity, gas saturation, or tuning effects caused by varying channel thickness. However, the well-tie is crucial in differentiating between true amplitude responses and tuning artifacts. Synthetic traces generated from well logs accurately reflect the seismic response of the reservoir. Comparing these traces with observed seismic amplitudes helps verify whether the stronger amplitudes are due to actual lithological changes or tuning effects. A well-calibrated time-depth relationship ensures that the seismic reflectors are accurately tied to reservoir zones, improving interpretation reliability.

### Seismic interpretation

*Seismic attributes*: Seismic attributes are mathematical functions applied to processed seismic data to highlight specific features or properties of the subsurface. Extracting various attributes such as amplitude variations with offset (AVO), spectral decomposition, and coherence can aid interpreters in identifying potential reservoirs, faults, and other geological structures (Schlumberger, 2011).

*Horizon picking*: Seismic interpreters manually pick seismic horizons that represent geological boundaries (e.g., top of a reservoir, base of an anomaly) based on seismic reflection patterns. This process creates a 3D seismic volume where each point represents a seismic reflection event and its corresponding two-way travel time (TWT) or depth. Accurate horizon picking is crucial for subsequent depth conversion and geological volume estimation.

*Integration with well data*: Well data, including well logs and horizon markers, were integrated with the interpreted seismic data. This process, known as a well-seismic tie, helps to calibrate the seismic response with the actual subsurface properties encountered in the wells. Well-seismic tie plays a vital role in validating seismic interpretation and improving the accuracy of depth conversion from two-way travel time to depth.

### Inversion and reservoir characterization

*Rock physics*: Rock physics, the bridge between seismic data and reservoir properties, plays a crucial role in deciphering the subsurface. It establishes quantitative relationships between physical rock properties, such as porosity, permeability, and mineral composition, and their measurable elastic properties^6,21^. Rock physics models, such as Gassmann’s equations, are employed to connect fluid substitution effects to elastic properties. The analysis assumes homogeneous, isotropic rock behavior for elastic wave propagation, neglecting anisotropic effects were minimal. Additionally, it is assumed that pore spaces are fully interconnected, allowing fluid mobility. The mineral composition is representative, primarily composed of quartz for sandstones (Table 1).

**Table 1:** Rock physics modeling key parameter ranges and values.

Lithology-fluid class	S-wave velocities (m/s)	Bulk density (g/cc)	P-wave velocities (m/s)	P-impedance ((m/s) ·(g/cm^3^))	V_p_/V_s_
Sand (water-saturated)	1200–1400	2.15–2.25	2400–2750	5160–6190	1.8–2.1
Sand (gas-saturated)	900–1300	2.00–2.24	1250–2200	2800–5500	1.3–1.7
Shale	1200–1800	2.2–2.4	3000–3500	1.8–2.1	1.7–2.3

By integrating rock physics principles with seismic data, geoscientists can transform seismic amplitudes and waveforms into meaningful reservoir parameters, enabling a more informed assessment of hydrocarbon potential. This study incorporates rock physics principles to establish relationships between seismic attributes derived from pre-stack simultaneous inversion and key reservoir properties within the Sapphire gas formation. This integration allows us to translate the seismic response into a quantitative understanding of the reservoir characteristics, ultimately leading to a more accurate and reliable evaluation of the resource potential.

*Inversion process*: Seismic inversion is a mathematical process that transforms seismic reflection amplitudes into quantitative estimates of elastic properties such as acoustic impedance or elastic moduli. These properties can be further related to rock and fluid properties relevant for hydrocarbon exploration (e.g., porosity, permeability, and fluid saturation)^1^. Seismic inversion provides valuable insights into the subsurface beyond the limitations of interpreting only the reflection amplitudes.

*Geo-body extraction*: Based on the interpreted seismic data and potential inversion results, geophysicists can delineate and extract 3D volumes representing geological bodies of interest, such as potential reservoir zones or fault blocks. This allows for a more quantitative assessment of these geological features.

*Reservoir properties*: By combining seismic interpretation, inversion results, and well data analysis, geophysicists can estimate and characterize reservoir properties such as porosity, fluid saturation, and net pay thickness. These properties are crucial for reservoir evaluation and development planning, enabling informed decisions regarding hydrocarbon exploration and production.

## Results and discussion

**Fig. 4:**
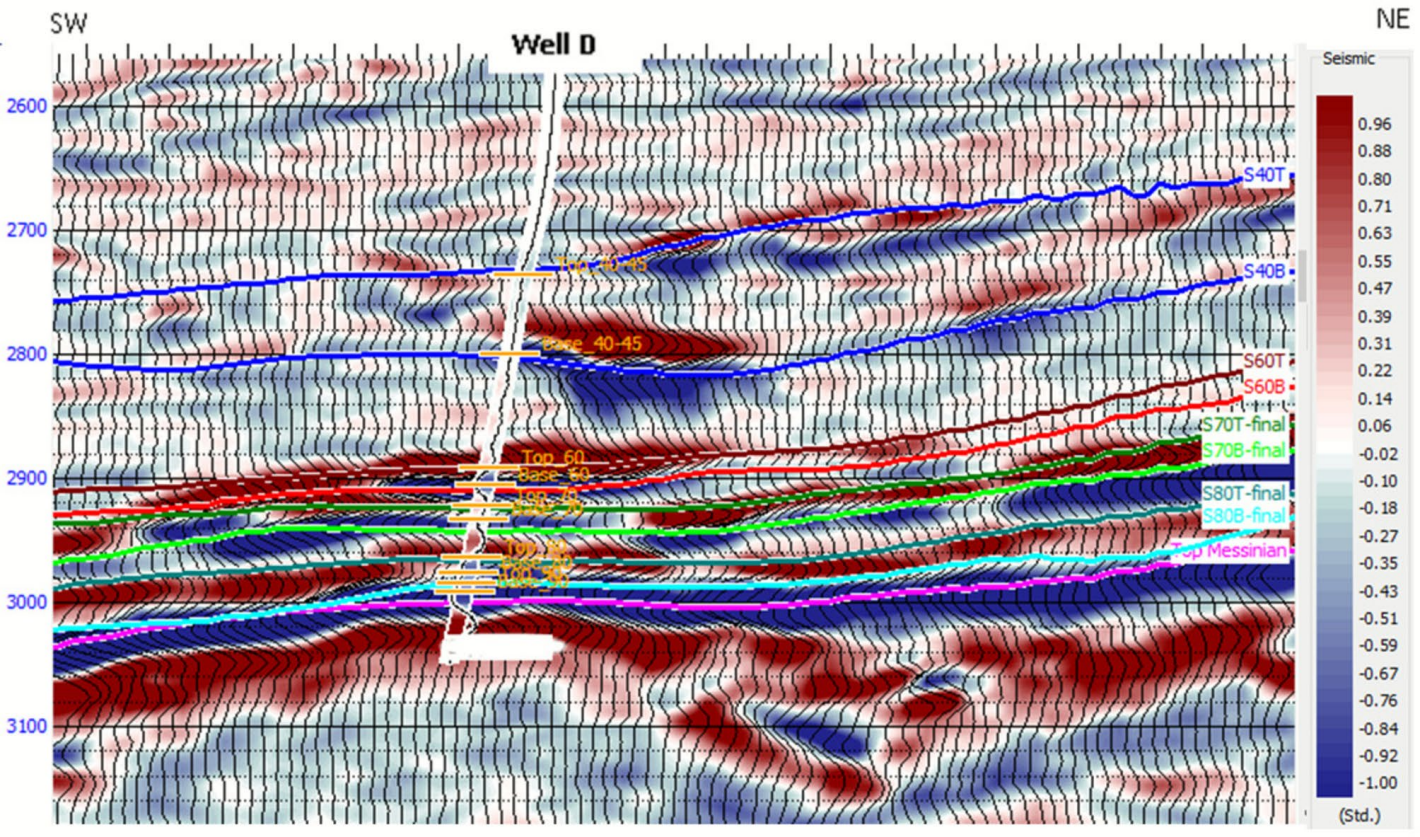
Illustrates the seismic interpretation for Sapphire horizons in Well D, showing the top-channel geological marker (soft kick) and base gas geological marker (hard kick).

**Fig. 5:**
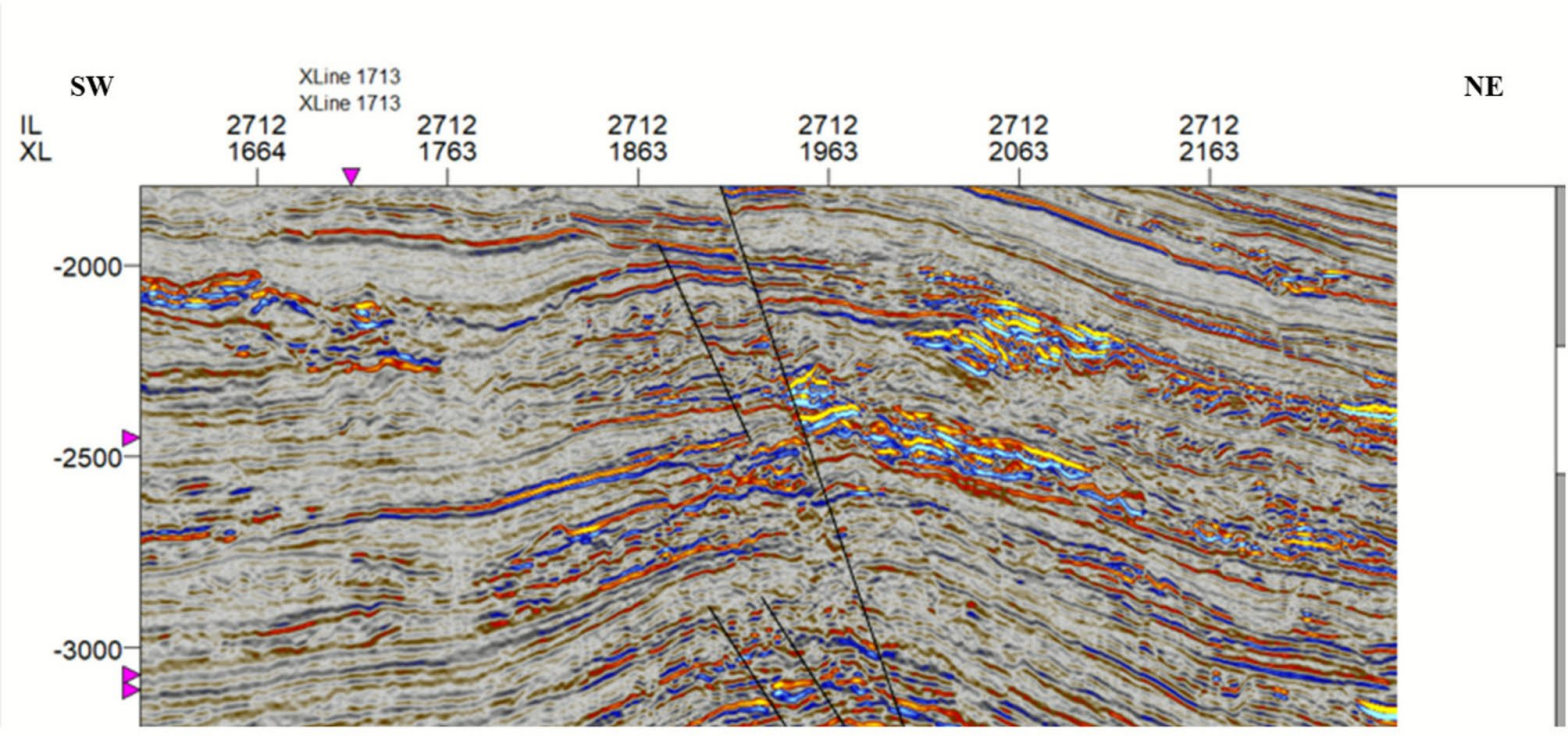
Illustrates the fault interpretation for a seismic section, highlighting some of the major faults within the study area.

Detailed seismic interpretation of the key seismic horizons was conducted over the Sapphire Field area of interest. Horizons were interpreted based on seismic events observed in the seismic-well ties as geological markers, which were defined as follows:

1. *Top channel/reservoir sand*: Mostly defined by a soft kick, that is, a reduction in acoustic impedance (AI) as the seismic rays cross the interface between the overlying shales (with larger values of AI) and gas-bearing sands (with smaller values of AI). This event mostly represents the top of the gas-bearing sand unit (i.e., the top-channel geological marker) (Fig. 4)
2. *Base gas*: Mostly defined by a hard kick, that is, an increase in acoustic impedance between the gas-bearing sands (with smaller values of AI) and the underlying units of either brine-bearing sands or shales (both with larger values of AI). This event mostly represents the gas–water-contact (GWC) or gas-down-to (GDT) geological markers (Fig. 4)
3. *Faulting*: Faulting in the Sapphire Field is considered a critical factor, likely responsible for field compartmentalization and impacting connected volumes and, therefore, accessible/recoverable reserves. Most faults in the field, particularly in the east, are part of the regional NDOA fault zone (Fig. 5) and are often of considerable length and displacement. Variance volume attributes were generated to reveal fault patterns over the area of interest (Fig. 7)

**Fig. 6:**
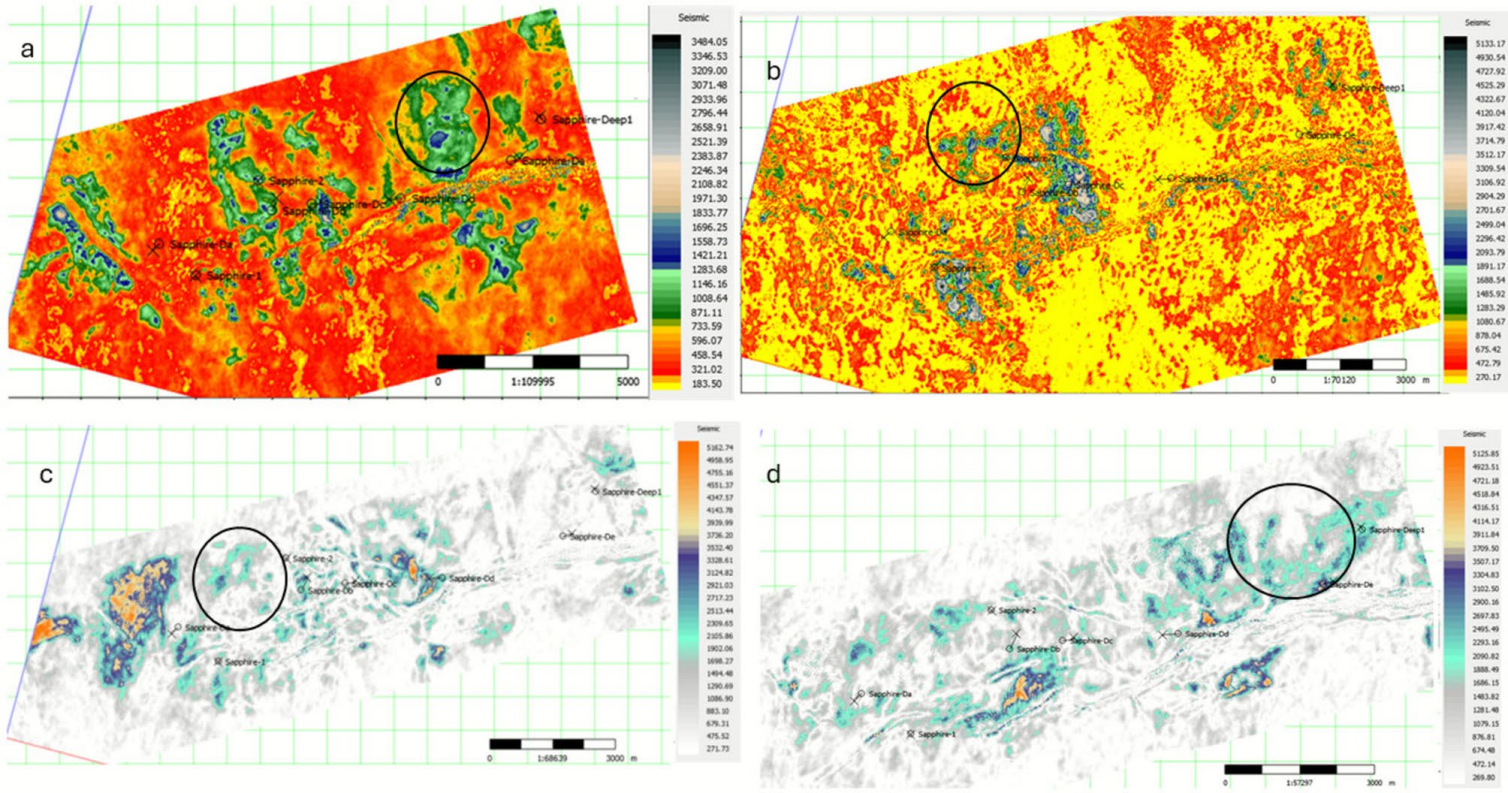
Horizon slices at four stratigraphic levels within the Sapphire gas-sand interval, displaying RMS amplitude (arbitrary units) with a color scale optimized to highlight lateral anomalies and channel geometries. (**a**) Top Sapphire-40, (**b**) Top Sapphire-60, (**c**) Top Sapphire-70, and (**d**) Top Sapphire-80. Well locations are marked with black dots. Circled zones indicate potential new hydrocarbon sweet spots, characterized by localized high-amplitude anomalies consistent with thickened or gas-charged sand channels. Scale bars indicate lateral distance; color intensity reflects relative RMS amplitude strength, not absolute physical units.

Understanding the intricate geometry and distribution of gas-sand channels within reservoirs is crucial for efficient resource exploration and production. Taking horizon slices at four different tops of a gas-sand unit, where the gas itself exhibits as a “soft kick” on seismic data, can yield valuable insights within the subsurface (Fig. 6).

By analyzing four horizon slices (Fig. 6), we can effectively “track” the evolution of the channels over time. This allowed us to map the lateral extent, sinuosity, and potential connectivity, thereby providing a more complete picture of the channel network. This information is invaluable for determining the flow paths of fluids within the reservoir and optimizing well placement.

Stacking, where channels overlap vertically, can significantly impact the reservoir volume and connectivity. Multiple-horizon slices enable the detection of stacked channels by observing their spatial relationships at different levels. This helps quantify the potential resource volume and assess the potential for crossflow between compartments.

Channels may terminate abruptly because of changes in the depositional environment or geological barriers. Studying multiple slices can reveal the termination patterns of the channels, providing essential information for predicting their effective flow boundaries and potential entrapment zones for hydrocarbons.

Channel thickness often varies laterally and vertically. By comparing multiple slices, we can map these variations and identify thicker zones within the reservoir that might hold greater gas potential. This knowledge can guide targeted drilling and potentially increase production efficiency.

Because the gas exhibits as a “soft kick” in seismic data, meaning that it appears as a subtle peak, careful interpretation is key. By analyzing the amplitude and character of the peak across different slices, we obtained the following insights.

1. A stronger peak might indicate a higher porosity and gas saturation within the channels, making them more attractive targets for production.
2. Subtle changes in the position of the peak across slices could potentially reveal the presence of fluid contact, further delineating the gas-filled zones within the reservoir.
3. Changes in the character of the peak, such as its sharpness or continuity, might suggest variations in the reservoir quality within the channels, highlighting potential challenges for production.

Taking multiple horizon slices of a gas sand channel reservoir unlocks this information. By interpreting the changes in channel morphology, stacking patterns, thickness variations, and the behavior of the “soft kick” gas response, we can gain a comprehensive understanding of this hidden resource. This knowledge empowers geologists and engineers to make informed decisions, optimize production strategies, and maximize the recovery of valuable hydrocarbons.

This study applies pre-stack seismic inversion using estimated velocity and density logs to an active field beneath the Nile Delta cone, with a focus on the Sapphire gas-bearing sand formation. Isolation is one of the major characteristics of inter-channel sandstone reservoirs in the study area. As a result, the extent of the reservoir is limited, and even a tight well placement can miss this prospect. Determining the location of a prospective reservoir can be misled in the traditional acoustic inversion of P-impedance (PI) alone.

The simultaneous pre-stack inversion of various elastic attributes can provide additional constraints for hydrocarbon exploration and lithology classification. An accurate quantitative seismic analysis using multiple elastic parameters is necessary to predict the distribution and geometry of reservoirs. This study demonstrates that even in areas with sparse well control and limited log availability, the pre-stack inversion procedure presented here can produce reliable results. Errors in the estimation process can be restricted to a small range by properly choosing the estimation method and testing its effectiveness in the local field.

The same workflow and approach can be applied to other fields similar to the Sapphire Formation for hydrocarbon exploration and depositional facies recognition and delineation.

### Seismic attribute extraction

Seismic amplitude variation plays a major role in identifying potential hydrocarbon reservoirs, helps to identify geological features, and indicates the presence of fluids. The 3-D seismic interpretation can be enhanced by interpreting seismic attributes such as amplitude, instantaneous frequency, reflection strength, and instantaneous phase polarity. Geological features, such as gross porosity and lithological contrast, were predicted using interpretation of seismic amplitude maps^22^.

**Fig. 7:**
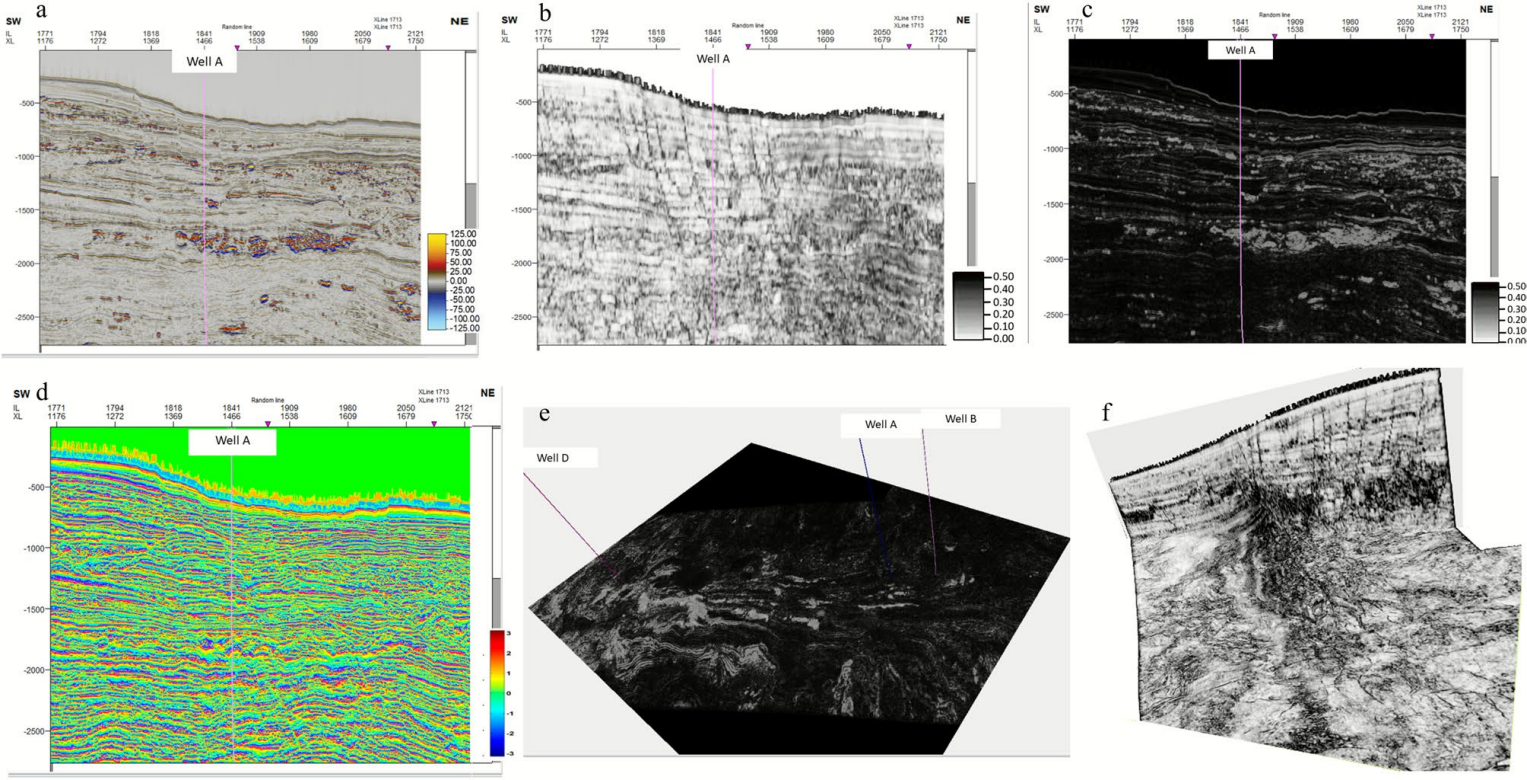
Multi-attribute seismic analysis at Well-C, demonstrating complementary roles in resolving structural and stratigraphic features. (**a**) Raw inline seismic section showing subsurface reflectivity; (**b**) Variance attribute highlighting lateral discontinuities associated with faults and channel margins; (**c**) Gradient magnitude accentuating sharp amplitude changes linked to lithological boundaries and channel edges; (**d**) Instantaneous phase enhancing reflector continuity to reveal subtle depositional geometries; (**e**) Gradient magnitude horizon slice mapping lateral variations in seismic response; (**f**) Variance horizon slice intersecting with crossline variance, illustrating 3D fault and channel geometry. All attributes are calibrated to well logs and integrated into the inversion-driven interpretation workflow.

Variance captures lateral discontinuities in seismic data, which are indicative of faults, fractures, or stratigraphic features, such as channels and pinch-outs. It highlights structural features and reservoir boundaries and is effective for delineating fault compartments and stratigraphic traps. This enhances structural mapping and aids in understanding the potential migration pathways and reservoir compartmentalization (Fig. 7).

The instantaneous phase indicates the precise “angle” of each peak and trough, essentially measuring how far it has progressed from the reference point. This allowed us to identify subtle changes in wave patterns, such as abrupt shifts in rock properties or the presence of faults. Faults, channels, and other discontinuities often disrupt the continuity of the wavefront, causing noticeable shifts in instantaneous phase. This attribute is invaluable for mapping these features and for understanding their impact on the subsurface (Fig. 7).

The magnitude of the gradient measures the rate of change in amplitude across a trace, highlighting subtle lithological variations and stratigraphic edges. It is sensitive to changes in acoustic impedance, such as transitions between shale and sand or fluid contact. This attribute enhances the definition of channel edges, levees, or clinoforms and is useful for detecting subtle stratigraphic features.

Gradient magnitude measures the rate of change in amplitude across a trace, highlighting subtle lithological variations and stratigraphic edges. It is sensitive to changes in acoustic impedance, such as transitions between shale and sand or fluid contacts. This attribute enhances the definition of channel edges, levees, or clinoforms and is useful for detecting subtle stratigraphic features.

### Geo-body extraction

**Fig. 8:**
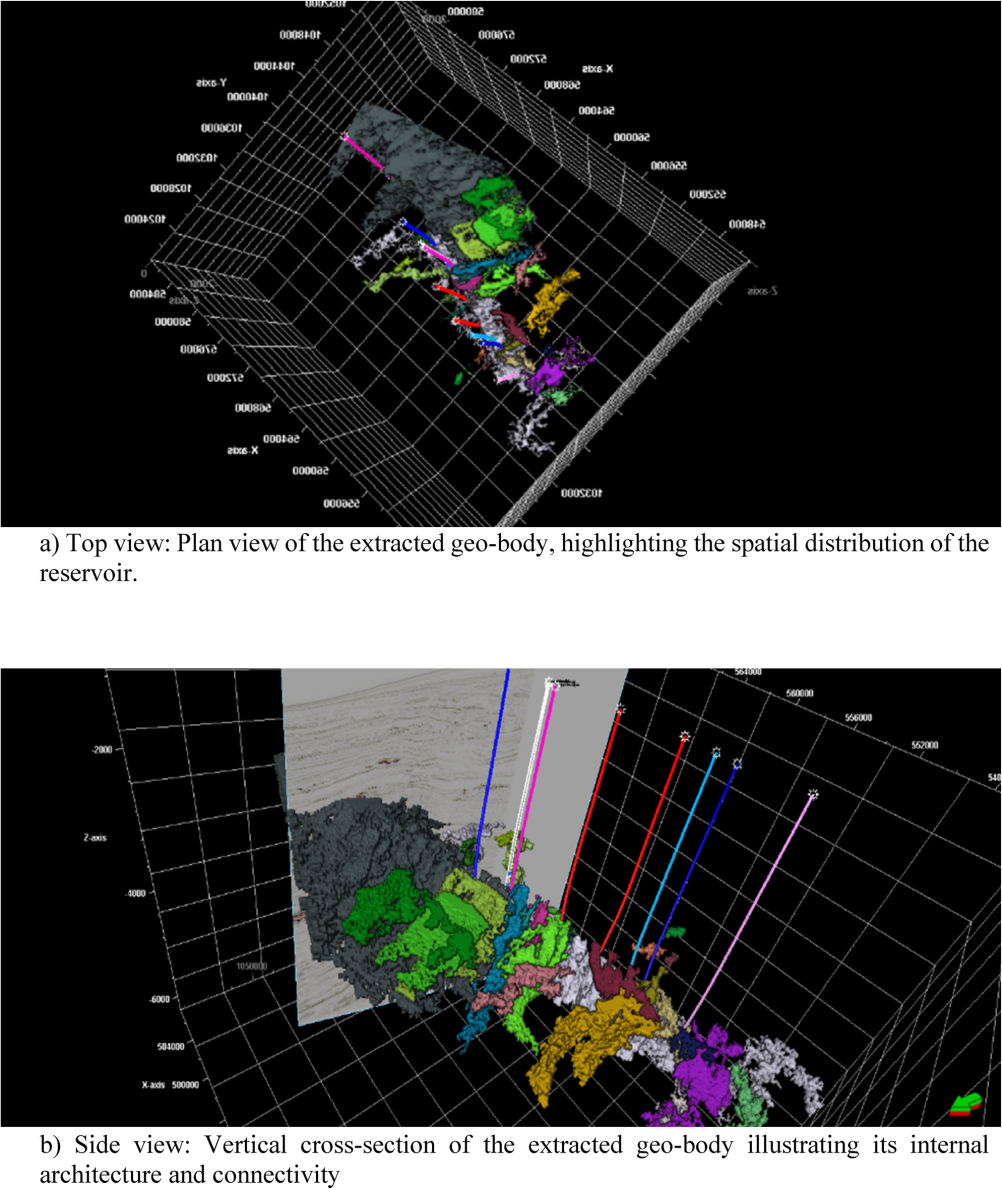
Seismic geo-body extraction for Sapphire Field.

**Fig. 9:**
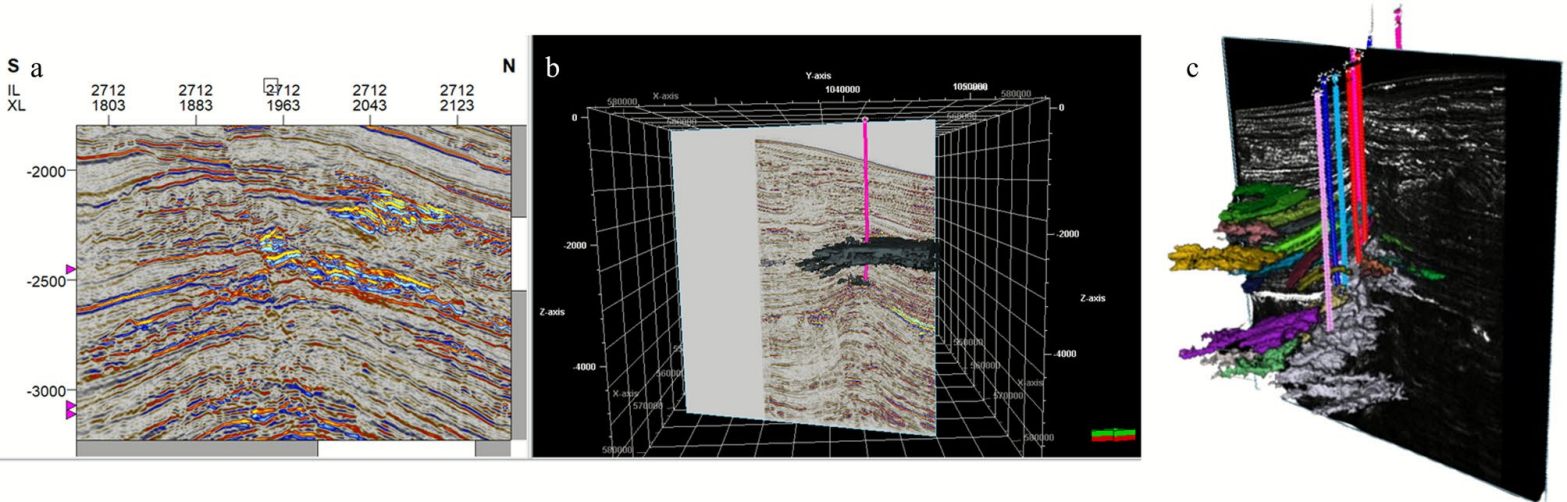
Multi-view visualization of geo-body extraction and validation in the Sapphire Field. (**a**) 2D seismic section intersecting the extracted geo-body, providing a vertical perspective of the reservoir; (**b**) 3D view of the extracted gas-sand channel system, illustrating its spatial extent and connectivity within the Pliocene turbidite sequence; (**c**) Intersection of the extracted geo-body with a gradient magnitude section, showing strong alignment between the geo-body boundaries and high-gradient zones—validating the accuracy of channel delineation through independent geometric criteria.

The key challenge of Sapphire Field development is to identify the connectivity between individual channel compartments for further field development. Figures 8 and 9 show the connectivity analysis applied to define the possible compartments for drilling. Connected pay sand geo-bodies were extracted from the RMS amplitude, which exhibited full frequency details, and the threshold of the RMS amplitude was tested iteratively, serving as an analog for geo-body extraction. Reservoir connectivity analysis showed that the pay sand volume was derived from geo-body extraction, which extends the sand bodies differently.

The emergence of automated horizon interpretation for extracted geo-bodies signifies a transformative leap in reservoir characterization, unlocking a multitude of advantages^23^. At its core, this technology automates the interpretation of horizons surrounding the geo-body, drastically streamlining workflows and minimizing the time and effort dedicated to this traditionally tedious task^24^. This newfound precision in delineating the surrounding layers facilitates the meticulous isolation of the geo-body within the seismic data, thereby unveiling its unique characteristic signature with unprecedented clarity^3^.

Furthermore, by allocating geo-body cells within a 3D grid, we can populate any property related to it, fostering a comprehensive understanding of its internal composition and behavior. This empowers reservoir engineers to optimize production strategies and predict fluid flow with new accuracy.

Moreover, sculpting horizons directly from the seismic volume surrounding the geo-body provide a vivid picture of the depositional environment. This provides crucial insights into reservoir formation and potential resource distribution, aiding targeted exploration efforts.

The primary focus in this work is Sapphire channels, where compartmentalization is more influenced by subtle depositional features rather than faults. Faults, although present, were not the dominant factors controlling the reservoir connectivity in this case. Most drilled wells target stratigraphic traps, limiting the opportunity to quantitatively validate fault-related compartmentalization. To address reservoir compartmentalization, simultaneous inversion results and key seismic attributes (e.g., variance and gradient magnitude) were analyzed to infer fault-related features indirectly.

Finally, well probes guided by interpreted horizons and geo-body characteristics minimize the drilling risk and cost. By accurately anticipating both lithological variations and structural complexities, engineers can identify the most promising drilling locations, save time and money, and avoid unproductive wells.

In essence, the automated horizon interpretation of extracted geo-bodies acts as a powerful key to unlocking knowledge within the reservoir. This refines our understanding, optimizes resource extraction, and minimizes risks, paving the way for a more efficient and productive future for the energy sector.

The major advantage of geo-body extraction is that it provides a gas–sand probability body, which highlights areas with better reservoir quality to be considered for well placement as part of the field development plan. The thin-bed contribution to reservoir connectivity was clarified by comparing the connected volumes produced by other techniques.

By interpreting the channels through the extracted geo-body, we gained a clearer and more holistic understanding of morphology, internal architecture, and interactions of the channel system with the surrounding geology. Data limitations and potential alternative interpretations should be considered to ensure comprehensive and nuanced analysis.

The margin between the gradient magnitude seismic attribute and the extracted gas-sand channels, represented by entirely white zones, offers a fascinating glimpse into subsurface secrets. When the seismic data exhibit sharp contrasts in amplitude, translated by gradient magnitude as high white values, we find the intricate pathways of these valuable channels etched within the rock. This perfect match validates the accuracy of the channel extraction and underlines the sensitivity of the gradient magnitude in identifying geological interfaces and boundaries. It is akin to unveiling a treasure map, with the white peaks on the gradient magnitude guiding us directly to the held within the gas-sand channels. This powerful synergy between seismic attributes and extracted geo-bodies paves the way for more precise reservoir characterization and targeted exploration, ultimately aiding the optimal extraction of these natural resources (Fig. 9).

### Rock physics analysis

The second stage aimed to identify the pore fluids present within the sapphire rocks. While drilling results and prior work confirm the presence of two primary pore fluid types (gas and brine) within the study area, the analysis specifically focuses on distinguishing gas-bearing sandstones from brine-saturated ones within the Sapphire sequence.

**Fig. 10:**
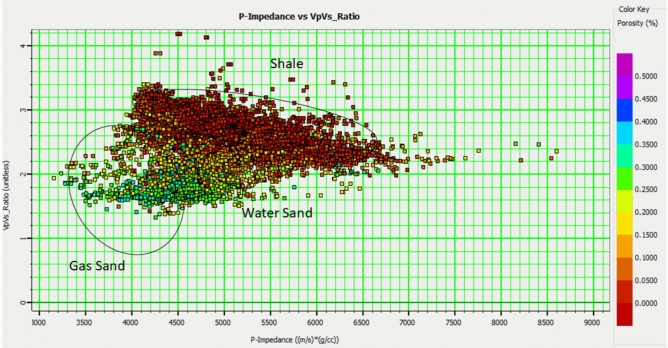
Well-based cross-plot between the V_p_/V_s_ ratio and P-impedance, color-coded with effective porosity, showing the distribution of points within the reservoir working interval for wells A, B, C, D, and E. This plot highlights the relationship between lithology, fluid content, and porosity in the Sapphire Reservoir.

**Fig. 11:**
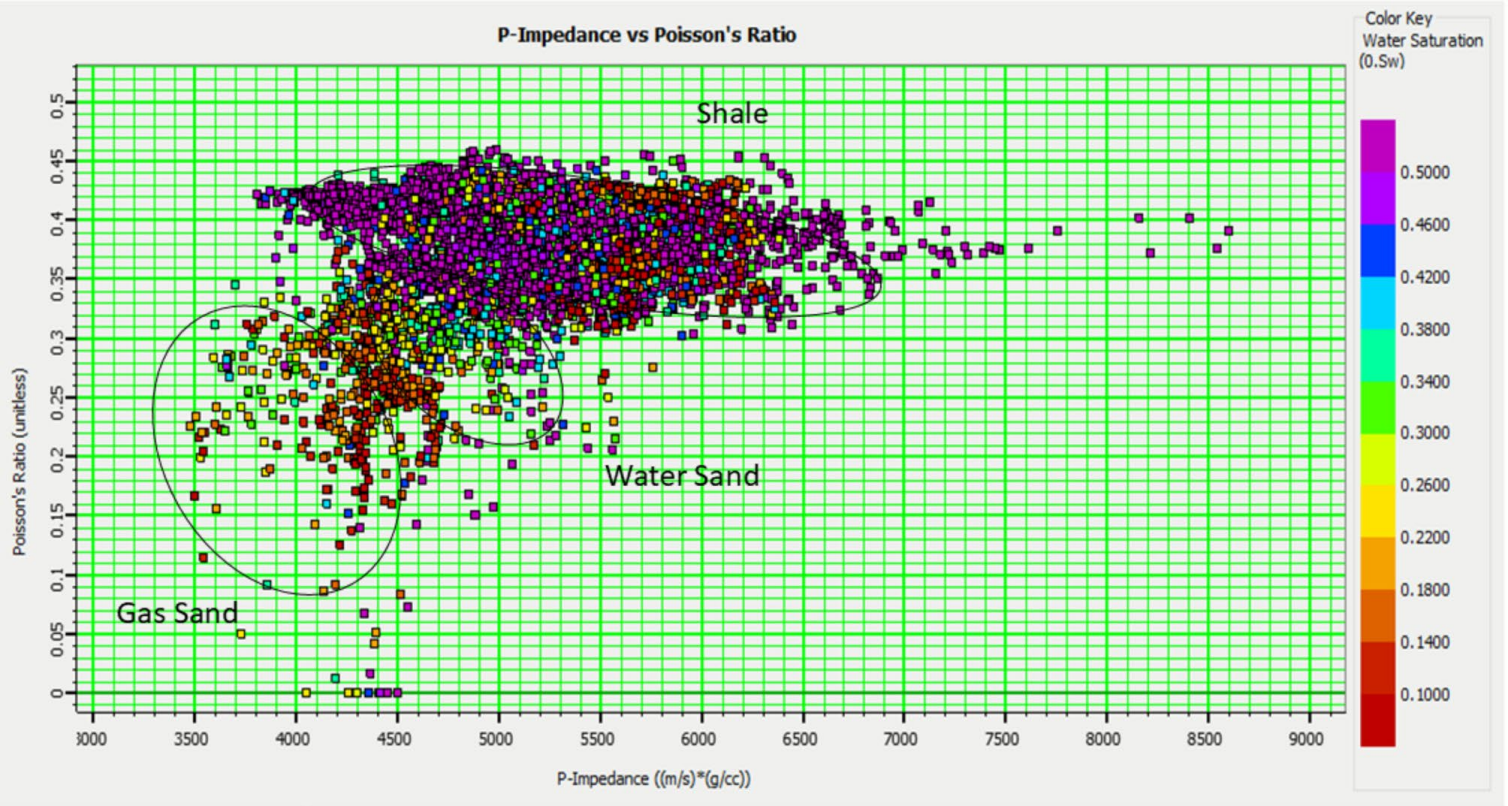
Well-based cross-plot between the Poisson ratio and P-impedance, color-coded with water saturation, showing the distribution of points within the reservoir working interval for wells A, B, C, D, and E. This plot provides insights into the fluid type and saturation within the reservoir.

Rock physics analysis serves as a crucial bridge between seismic, well log, and reservoir property data, enabling the interpretation of the seismic and logging responses of oil and gas layers and aquifers^6^. By constructing cross-plots of key elastic parameters, we can identify those that are most sensitive to lithology, physical properties, and fluid presence within the targeted area^25,26^. Figures 10 and 11 illustrate this approach, utilizing cross-plots of the P-to-S-wave velocity ratio (Vp/Vs) and P-wave impedance (PI) against the Poisson ratio and PI, respectively.

Analysis of these cross-plots, along with core and well log data, allowed us to differentiate reservoirs with desirable physical properties (e.g., high porosity) from the surrounding rocks. A specific focus is placed on distinguishing the physical properties of gas-containing layers from those of aquifers. Petrophysical analysis revealed that sandstone reservoirs with favorable properties exhibit low P-wave impedance, S-wave impedance (SI), density, and Lamé constant.

Key elastic parameters:

1. *P-Impedance*: Sensitive to lithological variations, such as sand vs. shale, owing to differences in bulk density and P-wave velocity. Highly responsive to changes in the fluid type (e.g., gas vs. brine).
2. *S-Impedance*: Strongly sensitive to lithology and porosity. Less influenced by fluid variations (S waves do not propagate through fluids).
3. *V*_*p*_*/V*_*s*_* Ratio*: Highly diagnostic of fluid type; gas-saturated rocks have significantly lower V_p_/V_s_ ratios than brine-saturated rocks.

Furthermore, the cross-plot of the V_p_/V_s_ ratio against P-impedance and the cross-plot of Poisson’s ratio against P-wave impedance (PI) (Figs. 13 and 14) effectively partitioned the reservoirs (Sapphire channel sandstone) into four distinct zones: gas zone , laminated zone, brine zone, and shale zone.

**Fig. 12:**
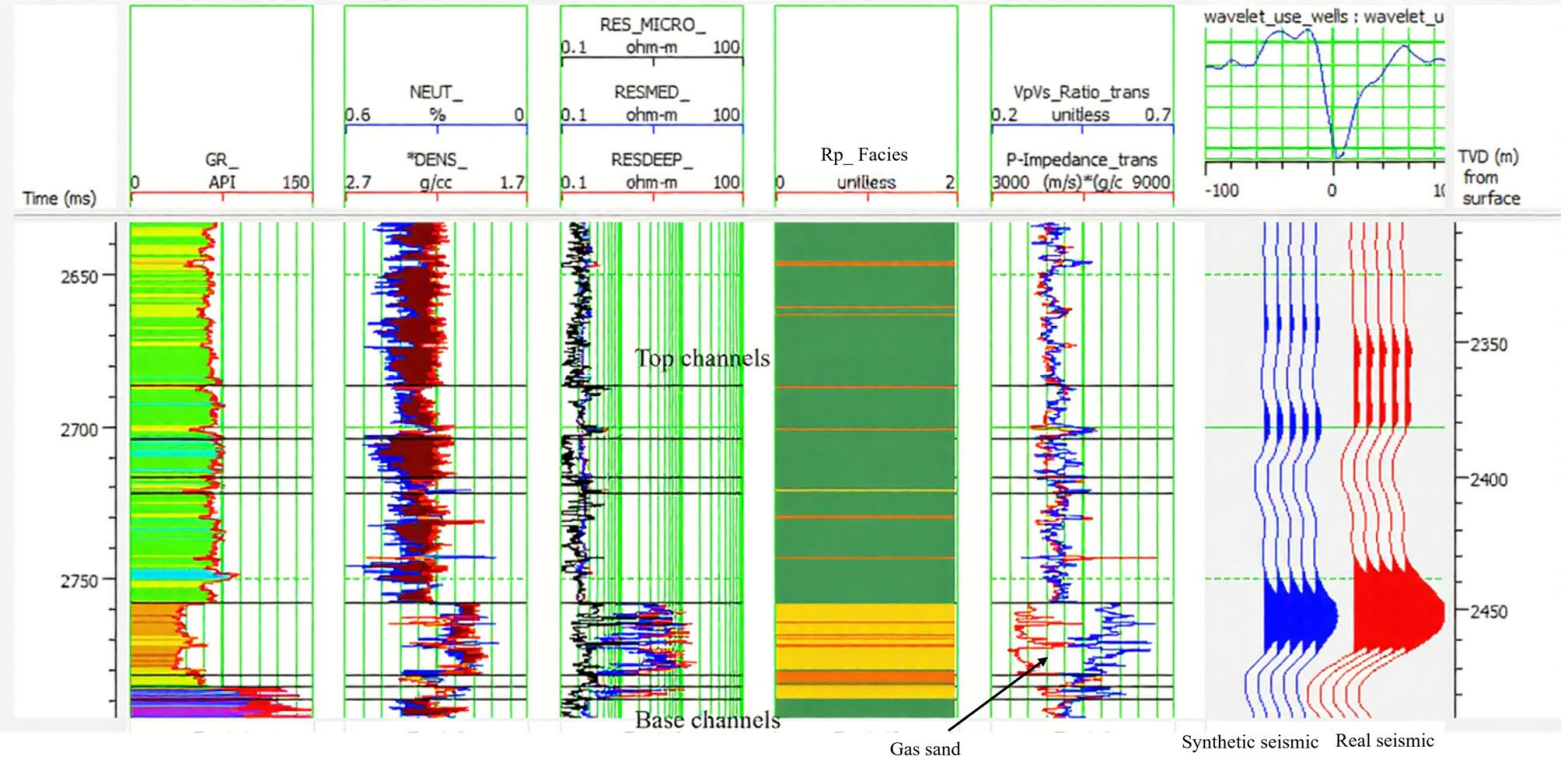
Rock-physics facies overlay on well logs, integrating petrophysical, elastic, and seismic data for reservoir characterization validation, displaying Track 1: Gamma Ray, Track 2: Neutron Density, track 3: Resistivity, Track 4: rock-physics facies (Green: Shale, Yellow: Gas-Sand, Orange: Laminated Shaly Sand), Track 5: P-impedance and V_p_/V_s_ ratio, and Track 6: Synthetic seismic trace (blue) generated from well logs compared to real seismic data (black), demonstrating a strong match at key reflectors. This figure integrates well log data with rock physics facies to validate the reservoir characterization.

This indicates that both acoustic impedances, along with the V_p_/V_s_ and Poisson ratios, offer critical insights into reservoir conditions in terms of both lithology and fluid content. Overlaying the well log data on the cross-plot allowed for a visual assessment of the accuracy of the facies classification (Fig. 12).

### Deterministic inversion results

Inversion results should be reviewed carefully to assess the reliability of using inversion products in quantitative reservoir modeling approaches. This can be performed in various ways, as described in this section. The elastic property cubes generated from this inversion include P-Impedance, S-Impedance, V_p_/V_s_ Ratio, and Density. The range of angles required for reliable density estimation is governed by seismic acquisition and processing parameters; however, inversion for density generally requires the utilization of far-angle ranges (30°–45°).

P-impedance and V_p_/V_s_ Ratio were considered the key elastic properties targeted from the pre-stack simultaneous inversion, based on the rock physics feasibility study in Section "Rock physics analysis".

Cross-plotting the inverted properties versus the well log properties, which were filtered to the seismic bandwidth, was a critical quality control step in the inversion process. This was done to check the match in the elastic property ranges between the inverted and measured logs. The degree of match can be defined by the gradients and correlation coefficients of the regression lines fitting the data points. For ideal inversion results, the fitting line gradient should be equal to one, while deviations from this value indicate either underestimation or overestimation of the inverted properties. In the present work, the gradient was estimated to be 0.8 for the P-impedance and 0.9 for the Vp/Vs ratio (Fig. 10).

**Fig. 13:**
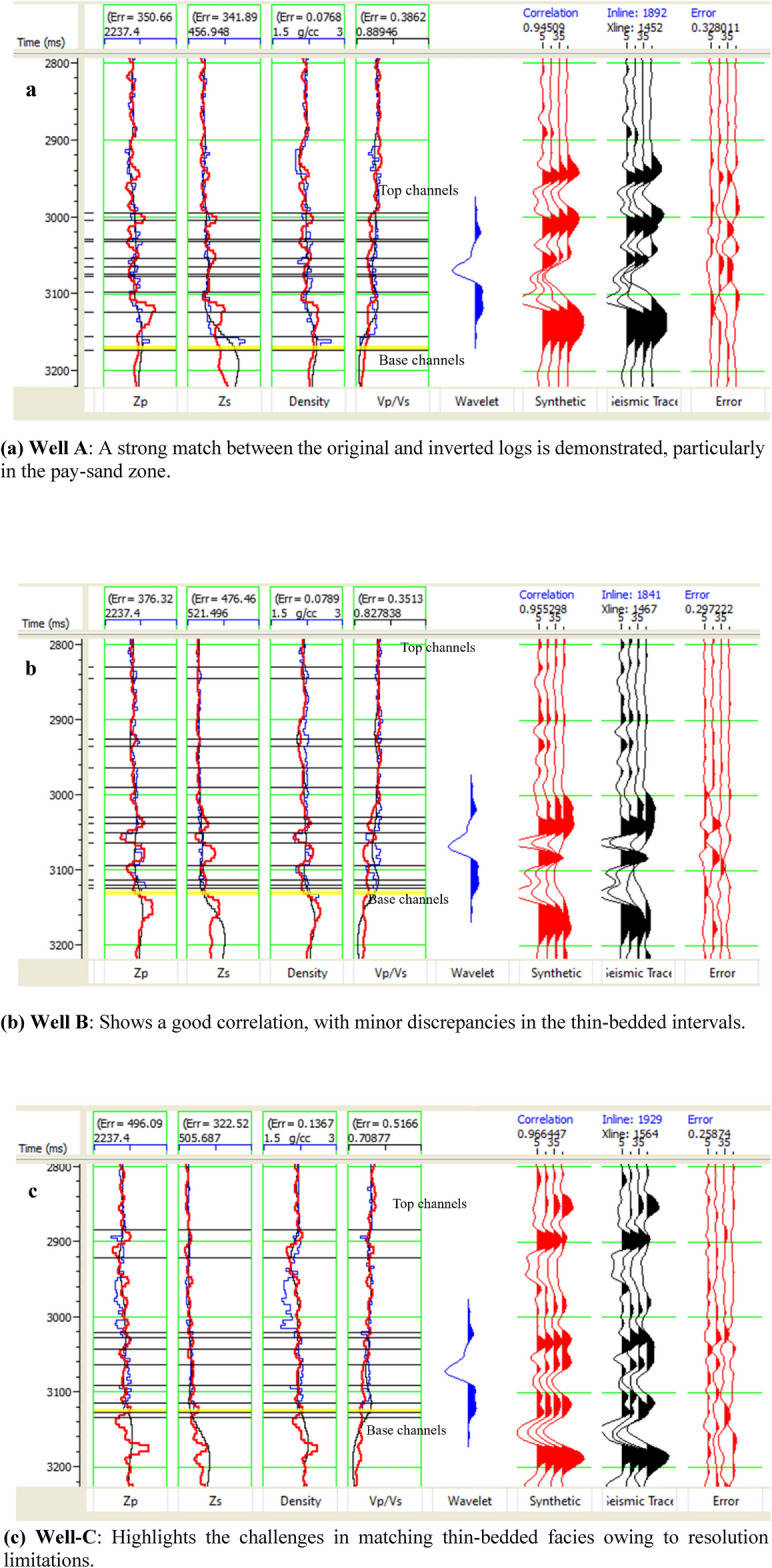
Inversion analysis for wells A, B, and C. The log panels from left to right display P-impedance, S-impedance, density, V_p_/V_s_ ratio, wavelet, synthetic, seismic traces, and error. The original logs are shown in blue, and the inverted logs are shown in red.

To objectively quantify the match between inverted and well-derived elastic properties, we computed Pearson correlation coefficients (R) and root-mean-square error (RMSE) between the inverted results and band-limited well logs for P-impedance at wells A, B, and C (Fig. 13). The results show strong agreement: Well A (R = 0.945, RMSE = 0.328), Well B (R = 0.955, RMSE = 0.297), and Well C (R = 0.966, RMSE = 0.259), with P-impedance in *(m/s)·(g/cm*^*3*^*)*. These metrics confirm high fidelity, particularly within the pay-sand zone. The slight waveform mismatch in Well C—despite high correlation—is attributed to thin-bed effects below tuning thickness (~ 9–20 m), consistent with phase distortions in laminated intervals. The blind-well test at Well D (Fig. 16) further validates the workflow, yielding comparable correlation (R ≈ 0.93) and low RMSE for P-impedance and V_p_/V_s_, confirming their reliability as key gas-sand discriminators.

The optimized parameterization of the inversion can be indicated by the match between the inversion results and well logs. Elastic property logs for all available wells were filtered to the seismic bandwidth by eliminating higher frequencies (above 60 Hz) compared with the inverted properties resulting from the deterministic seismic inversion.

1. P-impedance estimation is primarily influenced by reflections at near-normal incidence angles (0°–20°), where the amplitudes are dominated by the P-wave energy. The accuracy remained stable even as the angle increased, although the contribution of the P-wave energy diminished at steeper angles. P-impedance exhibits the highest reliability among the inverted parameters, as it depends primarily on robust low-angle amplitudes that are less affected by noise and wavelet inconsistencies.
2. S-impedance becomes increasingly reliable at moderate to high incident angles (20°–30°). At these angles, the reflection coefficients are sensitive to S-wave velocity contrasts, allowing for better resolution of lithology and fluid effects. However, the presence of noise at these angles requires careful data conditioning. S-impedance becomes a reliable parameter only at angles where the seismic data contain sufficient information about shear wave variations, typically exceeding 20°.
3. Density estimation is the most challenging aspect of seismic inversion because of its weak influence on reflection coefficients at lower angles. It becomes meaningful only at mid-high incident angles (30°–45°), where variations in reflection amplitudes are more sensitive to density changes^19^.

**Fig. 14:**
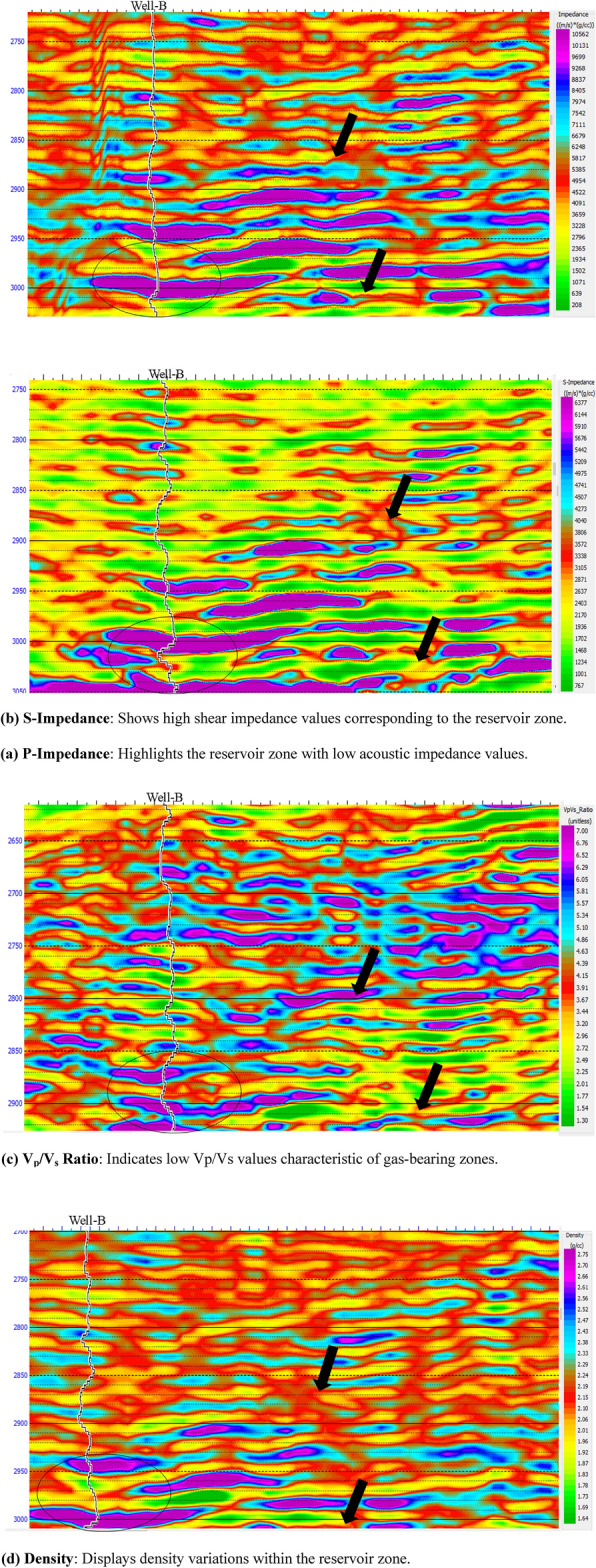
Inverted elastic attributes. Ellipses indicate the reservoir zone, represented by low V_p_/V_s_ and high S-impedance values. The inserts show the acoustic impedance, shear impedance, V_p_/V_s_ ratio, and density logs with a similar bandwidth for comparison with the inversion results, demonstrating a correlation of approximately 95. The arrows indicate zones with anomalous values similar to those of the reservoir.

Figure 14 shows the good match between the filtered and inverted P-impedance at Well A, Well B, and Well C. The better the quality of the gas sand, the lower the impedance of the layer, and vice versa. The aquifer layers were expected to be located down-dip of the structural closure and were considered acoustically harder than gas sand and shale.

Reservoir sweet spots can also be identified by low Vp/Vs values, as shown in Fig. 13. Well-C encountered thin-bedded facies, which fell below the deterministic inversion resolution, resulting in a less accurate match between the measured Vp/Vs log and the inverted property at Well-C compared to Well-A.

The pre-stack seismic inversion in this study aimed to overcome this resolution issue and provide the ability to map relatively thinner facies. Once the inverted elastic properties are investigated and quality-controlled, they can be used to derive facies and fluid probabilities to further quantify the results.

To ensure quality, it is critical to analyze the inversion results from real data before applying the inversion method to the entire volume. Both Well-A, Well-B, and Well-C show a good correlation between the inverted and original logs, especially for the pay sand zone, where V_p_/V_s_ and S-Impedance show distinct values from the surrounding layers (Fig. 13).

A comparison between the inversion results and the well logs shows a good match, particularly in key elastic properties such as the P-impedance and V_p_/V_s_ ratio, which align well with the gas-saturated zones and lithological boundaries. However, minor discrepancies were observed in thin-bedded areas, likely due to the seismic resolution limits or tuning effects. The overall consistency across multiple well locations enhance confidence in the inversion results; however, further refinement of the low-frequency model and wavelet extraction could improve accuracy, especially for more complex zones.

The mismatch in the upper part of the Sapphire channels is a result of the interbedded thin layers. Although the inverted elastic parameters in this window may not accurately reflect the thin layers, the general trend matches well with the computed log values, and the inversion pay sand zone is not affected. The simultaneous inversion algorithm was applied to the entire data volume, as shown along the arbitrary line crossing all five wells (Fig. 14). It is crucial to note that the inversion was performed in the time domain rather than depth.

Both the S-Impedance, P-Impedance, and Density attributes show significant anomalies, whereas the Vp/Vs attribute shows considerably low values. However, the exact extent of the area cannot be determined with confidence because of the presence of similar values in the surrounding regions. The significantly high S-impedance, low P-impedance, low density, and low Vp/Vs represent a possible gas-saturated zone, which can also be observed in up-dipping regions, as marked by the arrows in Fig. 14.

**Fig. 15:**
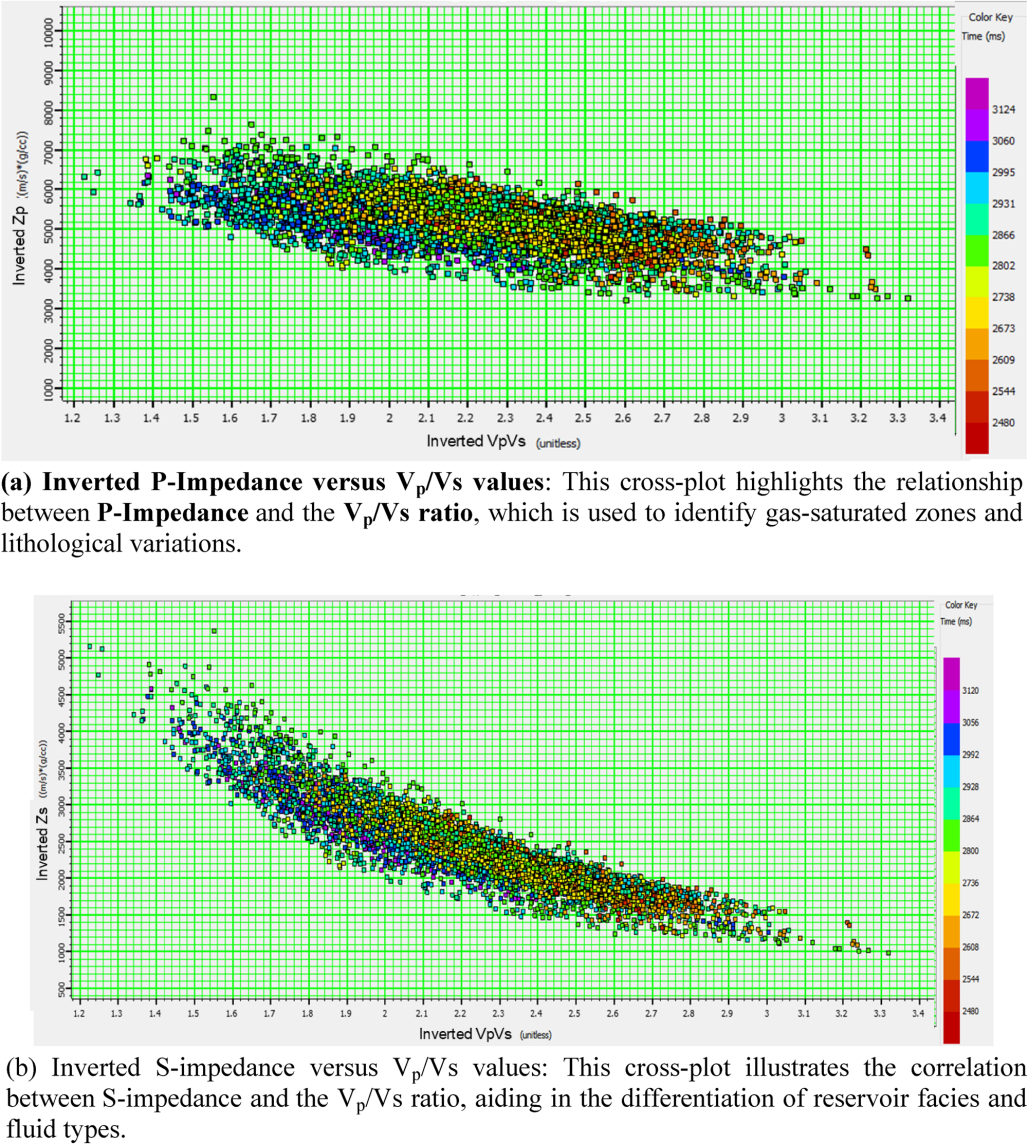
Relationship between inverted impedance and V_P_/V_S_ values derived from the simultaneous inversion.

To map the reservoir area, we cross-plotted the inverted Vp/Vs volume versus the inverted P-impedance volume (Fig. 15), similar to the rock physics analysis conducted on wells. The volume data show a distinctive cluster of gas sand and a background trend. An area was selected using the range obtained from the analysis of nearby wells, where the Vp/Vs ranged from 1.5 to 1.65 and the S-impedance ranged from 16,000 to 21,500 (Fig. 15).

**Fig. 16:**
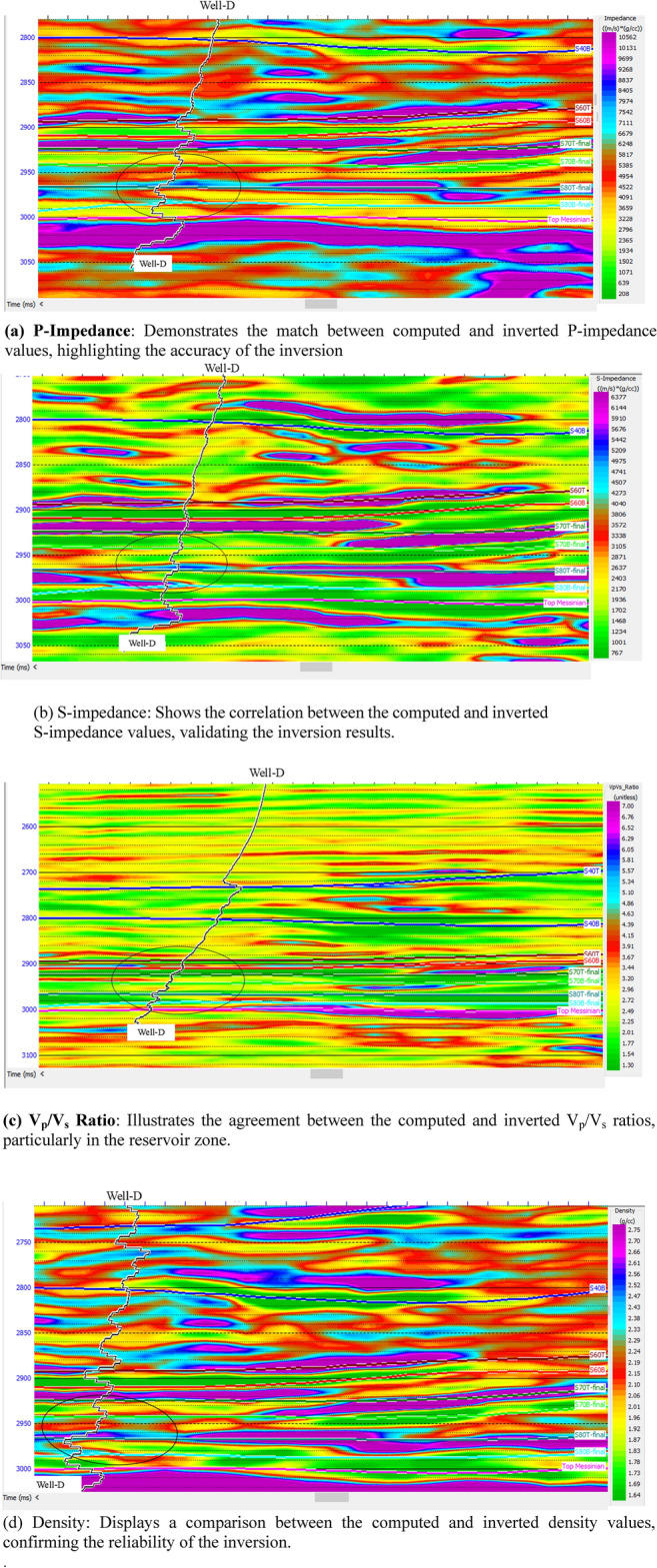
Inversion quality control with Well D.

The blind well, Well-D, was used to test the accuracy of the inversion results (Fig. 16). Traces from each inverted attribute volume were extracted at the well location and overlapped with the computed logs. The error was calculated by subtracting the inverted values from the computed values. The error analysis results suggest that the parameters of the simultaneous inversion are reliable and can be used for reservoir characterization with confidence.

The reservoir area, represented by high S-impedance and low V_p_/V_s_, correlates well with the pay sand zone in Well D, confirming the reliability of the inversion results (Fig. 16).

### Limitations and challenges

While the inversion workflow yielded robust results, several inherent limitations must be acknowledged. The seismic data is bandlimited to 10–60 Hz, with a dominant frequency of approximately 30–40 Hz in the reservoir interval. Given P-wave velocities derived from well logs (1250–2750 m/s, calculated from P-impedance of 2800–5500 (m/s)·(g/cm^3^)) and density of 2.0–2.24 g/cm^3^), the practical tuning thickness ranges from 9 to 20 m. Beds thinner than this cannot be resolved as discrete layers and instead produce vertically averaged elastic responses—evident in the laminated intervals at Well-C (Fig. 13c) and minor stratigraphic mismatches in attribute maps (Fig. 12). This underscores that the workflow reliably captures elastic trends across seismic horizons rather than individual sub-resolution beds. Although our angle stacks span 0°–50°—a range sufficient to support simultaneous inversion of P-impedance, S-impedance, and density—the signal-to-noise ratio decreases with increasing incident angle due to geometric spreading, attenuation, and multiple interference. Since density estimation relies heavily on far-angle data (30°–45°), it remains the least reliable inverted parameter, as confirmed by lower correlation in blind-well tests (Fig. 16d), with implications for derived properties such as porosity. The inversion assumes a single deterministic wavelet with a constant phase of –79° applied uniformly across all angle stacks; while validated by strong well ties (correlation = 0.913), real wavelets may exhibit subtle angle-dependent variations due to attenuation or processing, potentially introducing minor inconsistencies in high-angle responses. Additionally, uncertainties in the low-frequency model (< 10 Hz)—constructed from kriged well logs and constrained by check-shot surveys—can introduce systematic biases in absolute impedance values, though relative trends remain robust for lithology–fluid discrimination. Despite these constraints, the blind-well test at Well-D (Fig. 16) confirms that P-impedance and V_p_/V_s_, our primary discriminators for gas-bearing sands, are reliable within the resolution, bandwidth, and angular limits of the data.

### Broader implications and transferability of the workflow

Beyond the Sapphire Field, this study offers a transferable framework for characterizing thin-bedded, gas-charged sand channels in highly compartmentalized deepwater clastic systems. The workflow advances current practice in three keyways. First, unlike AI-driven bright-spot detection^23,27^ or inversion approaches developed for carbonate reservoirs^26^, our method is physics-based, clastic-optimized^28^, and validated in a Pliocene turbidite setting where thin beds and structural complexity challenge conventional interpretation. Second, we synergistically integrate simultaneous pre-stack inversion with gradient magnitude attributes—enabling cross-validation of geo-body boundaries through independent physical (elastic impedance contrasts) and geometric (seismic waveform discontinuity) criteria (Fig. 12). Third, we establish robust, quantitative elastic thresholds for gas-sand identification (P-impedance < 18 *(m/s)·(g/cm*^*3*^*)*; Vp/Vs < 1.65), validated across five wells, including one held-out blind well excluded from inversion calibration.

This approach directly addresses critical industry challenges: it reduces dry-hole risk in stratigraphic traps, enables precision targeting of infill wells in fault-compartmentalized fields, and provides a reproducible template for analogous settings. Immediate applications include other Nile Delta fields such as Burullus and Baltim, which share similar Pliocene–Pleistocene deepwater depositional systems. More broadly, the framework is adaptable to any clastic deepwater province where thin-bed resolution, sparse well control, and fault-driven compartmentalization limit reservoir predictability—from the Gulf of Mexico and West Africa to Southeast Asia. By anchoring seismic interpretation in first-principles rock physics while leveraging high-resolution geometric attributes, this study bridges the gap between site-specific case analysis and generalizable subsurface prediction.

## Conclusion

This study demonstrates that integrating pre-stack simultaneous inversion with seismic attribute analysis significantly enhances reservoir facies identification in thin-bedded, compartmentalized clastic systems. Applied to the Pliocene turbidite reservoir of the Sapphire Field (offshore Mediterranean, Egypt), the workflow successfully delineates isolated gas-sand channels by combining rock-physics-guided elastic thresholds (P-impedance < 18 *(m/s)·(g/cm*^*3*^*)*; V_p_/V_s_ < 1.65)—validated across five wells including a blind test—with attribute-enhanced geo-body extraction. Key advances include: (1) a synergistic integration of simultaneous inversion and gradient magnitude to cross-validate channel geometry using independent elastic and geometric criteria; (2) robust, quantitative criteria for gas-sand discrimination under sparse well control; and (3) the recognition that stratigraphic architecture—not faulting—dominates reservoir compartmentalization in this setting.

Unlike conventional post-stack inversion or qualitative amplitude interpretation, this physics-based approach reduces exploration uncertainty, identifies bypassed pay zones, and enables optimal well placement. Critically, the methodology is transferable to analogous deepwater clastic systems globally—from the Nile Delta to the Gulf of Mexico and West Africa—providing a reproducible framework for reservoir characterization where thin beds and limited well data challenge traditional workflows. By bridging rock physics, seismic inversion, and geometric attributes, this study advances quantitative interpretation beyond site-specific analysis toward generalizable subsurface prediction.

## Data Availability

The datasets generated and/or analyzed during the current study are not publicly available due to the confidentiality agreement signed between the EGPC (the Egyptian General Petroleum Corporation), Shell-Egypt and the authors but are available from the corresponding author upon reasonable request and with permission of the EGPC (the Egyptian General Petroleum Corporation) and Shell-Egypt.
